# PTEN suppresses the oncogenic function of AIB1 through decreasing its protein stability via mechanism involving Fbw7 alpha

**DOI:** 10.1186/1476-4598-12-21

**Published:** 2013-03-21

**Authors:** Chunhua Yang, Shujing Li, Miao Wang, Alan K Chang, Ying Liu, Feng Zhao, Liyun Xiao, Lin Han, Dao Wang, Shen Li, Huijian Wu

**Affiliations:** 1 School of Life Science and Biotechnology, Dalian University of Technology, 2 Ling Gong Road, Dalian 116024, China

**Keywords:** PTEN, AIB1, Transcriptional activity, Ubiquitination, Fbw7 alpha, Breast cancer

## Abstract

**Background:**

Phosphatase and tensin homologue deleted on chromosome 10 (PTEN) is a phosphatase having both protein and lipid phosphatase activities, and is known to antagonize the phosphoinositide 3-kinase/AKT (PI3K/AKT) signaling pathway, resulting in tumor suppression. PTEN is also known to play a role in the regulation of numerous transcription factors. Amplified in breast cancer 1 (AIB1) is a transcriptional coactivator that mediates the transcriptional activities of nuclear receptors and other transcription factors. The present study investigated how PTEN may regulate AIB1, which is amplified and/or overexpressed in many human carcinomas, including breast cancers.

**Results:**

PTEN interacted with AIB1 via its phophatase domain and regulated the transcriptional activity of AIB1 by enhancing the ubiquitin-mediated degradation of AIB1. This process did not appear to require the phosphatase activity of PTEN, but instead, involved the interaction between PTEN and F-box and WD repeat domain-containing 7 alpha (Fbw7α), the E3 ubiquitin ligase involved in the ubiquitination of AIB1. PTEN interacted with Fbw7α via its C2 domain, thereby acting as a bridge between AIB1 and Fbw7α, and this led to enhanced degradation of AIB1, which eventually accounted for its decreased transcriptional activity. At the cell level, knockdown of PTEN in MCF-7 cells promoted cell proliferation. However when AIB1 was also knocked down, knockdown of PTEN had no effect on cell proliferation.

**Conclusions:**

PTEN might act as a negative regulator of AIB1 whereby the association of PTEN with both AIB1 and Fbw7α could lead to the downregulation of AIB1 transcriptional activity, with the consequence of regulating the oncogenic function of AIB1.

## Background

*Phosphatase and tensin homologue deleted on chromosome 10 (PTEN)* was originally discovered as the tumor suppressor gene frequently lost on chromosome 10q23 [1]. PTEN is a phosphatase having both protein and lipid phosphatase activities. It is well-defined as a tumour suppressor that plays a critical role in cell survival and cell death [2]. A high frequency of mutation in *PTEN* is associated with the development of various types of human diseases [3], including glioblastomas [4], prostate cancers [5], and endometrial carcinomas stimulated by tamoxifen [6,7]. The complete loss of PTEN is also a common event in breast cancers that are caused by *breast cancer 1 (BRCA1)* deficiency [8]. PTEN has a phosphatase (PPase) domain, which specifically dephosphorylates phosphoinositide-3,4,5-triphos-phate (PIP3), a potent activator of AKT. It therefore acts as a negative regulator of the PI3K/AKT signaling pathway, which is specifically involved in cell growth, apoptosis, transcription and cell migration. In addition to its phosphatase domain, PTEN also has a putative C2 regulatory (C2) domain and a C-terminal tail (Tail) containing two PEST homology regions that also play important roles in regulating its function [9,10]. For example, PTEN can associate with the centromere by docking onto centromere protein C (CENP-C), a centromeric binding protein, resulting in the maintenance of chromosomal stability [11]. A recent study has shown that PTEN can interact with anaphase-promoting complex/cyclosome (APC/C), an E3 ubiquitin ligase, and promote its association with cadherin 1 (CDH1), thereby enhances the tumor-suppression activity of the APC-CDH1 complex [12]. In both cases, the phosphatase activity of PTEN is not required.

Amplified in breast cancer 1 (AIB1), also known as SRC-3/ACTR/RAC3/Ncoa3, is a member of the p160 family, which also includes SRC-1 and SRC-2/GRIP1. AIB1 was initially found to be amplified in breast cancer [13], but was later also found to be amplified in other cancers [14], including ovarian cancers [15,16], endometrial carcinomas [17], pancreatic cancers [18] and prostate cancer [19]. In mice models, AIB1 overexpression is linked to high frequency of tumorigenesis in mammary gland pituitary, uterus and lung [20,21], and AIB1 knockdown would lead to inhibition of mammary gland tumorigenesis induced by oncogene *HER2/neu*[22]. These observations indicate that AIB1 plays a key role in the development and progression of several different cancers. AIB1 acts as a transcriptional coactivator of nuclear receptors such as estrogen receptor alpha (ERα), and recruits secondary coactivators, including p300/CBP to facilitate the transcription of target genes [23]. Moreover, AIB1 also plays a role in epidermal growth factor receptor (EGFR) signaling and insulin-like growth factor (IGF) signaling [24].

AIB1 is tightly regulated, especially by post-translational modification, which includes phosphorylation, acetylation, methylation, ubiquitination and sumoylation [25-27]. Post-translational modification of AIB1 can either up-regulate or down-regulate its protein or activity level. For examples, dephosphorylation of AIB1 by several phosphatases pyridoxal phosphate phosphatase (PDXP), protein phosphatase 1 (PP1), and protein phosphatase 2A (PP2A) can suppress its transcriptional activity [28], whereas ubiquitination of AIB1 can lead to its degradation [29]. Among the three enzymes (E1, E2 and E3) that catalyze the ubiquitination of proteins, only E3 ubiquitin ligases physically interact with their substrates, and therefore confer some degree of specificity. Several E3 ubiquitin ligases are known to associate with the ubiquitination of AIB1, and these are E6-associated protein (E6-AP), F-box and WD repeat domain-containing 7 alpha (Fbw7α) and speckle-type POZ protein (SPOP) [30-32]. Among them, Fbw7α has been widely investigated. It is a classical E3 ubiquitin ligase of AIB1, and it controls numerous cellular processes, including cell-cycle progression, cell proliferation and differentiation through degrading a set of well-known oncoproteins such as c-myc and cyclin E in addition to AIB1 [33].

In this study, we showed that PTEN could act as a negative regulator of AIB1 through decreasing its protein stability, leading to suppression of its transcriptional activity and oncogenic function. We also presented evidence to show that such regulation of AIB1 by PTEN occurred via a mechanism that involved Fbw7α.

## Results

### PTEN decreases AIB1 protein level via promoting its degradation

**Figure 1:**
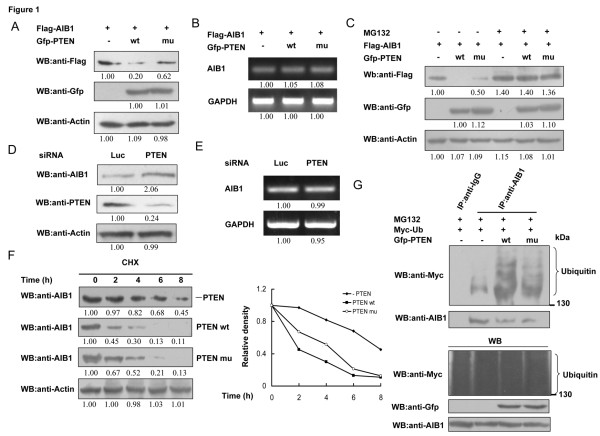
**Effect of PTEN on the degradation of AIB1. (A)** COS-7 cells transfected with Flag-tagged AIB1 and Gfp-tagged wild-type (wt) or G129R mutant (mu) PTEN were collected 24 h after transfection and subjected to western blot analysis with the indicated antibodies. **(B)** COS-7 cells were transfected with Flag-tagged AIB1 and Gfp-tagged wt or mu PTEN, and then subjected to reverse-transcription PCR analysis 24 h after transfection. **(C)** COS-7 cells transfected with Flag-tagged AIB1 and Gfp-tagged wt or mu PTEN were treated with or without 10 μM MG132 for 8 h. The cells were then collected and subjected to western blot analysis with the indicated antibodies. **(D)** MCF-7 cells transfected with siPTEN or control plasmid (siLuc) were collected and subjected to western blot analysis with the indicated antibodies 24 h after transfection. **(E)** MCF-7 cells transfected with siPTEN or control plasmid (siLuc) were collected 24 h after transfection and subjected to reverse-transcription PCR analysis. **(F)** MCF-7 cells transfected with or without Gfp-tagged wt or mu PTEN were treated with 10 μg/ml cycloheximide (CHX) for different periods of time (0, 2, 4, 6, 8 h) before subjected to western blot analysis to detect the change in AIB1 protein level. The graph shows the relative intensity of the AIB1 bands at the different time points. **(G)** MCF-7 cells transfected with Myc-tagged Ub and Gfp-tagged wt or mu PTEN were treated with 10 μM MG132 for 8 h. The cells were collected and then subjected to immunoprecipitation with anti-IgG or -AIB1 antibody followed by western blot analysis with anti-Myc antibody.

Given that PTEN could induce apoptosis in a variety of cell types, including breast cancer cells, and that AIB1 is an oncogenic protein which is often overexpressed in breast cancer cells, we speculated that there could be a potential connection between PTEN and AIB1 signaling pathways. First we examined whether there is a causal relationship between PTEN and AIB1 at the protein level by transfecting COS-7 cells with Flag-tagged AIB1 and Gfp-tagged wild-type PTEN or its mutant (G129R) deficient in both lipid and protein phosphatase activities [34] and compared the levels of AIB1 protein in these cells using western blot. Expression of wild-type or mutant PTEN resulted in reduced AIB1 protein level, with wild-type PTEN causing a stronger reduction (Figure 1A). Reverse-transcription PCR analysis showed that both wild-type and mutant PTEN had no effect on the level of AIB1 mRNA (Figure 1B), suggesting that the reduced level of AIB1 protein caused by PTEN was due to a change in AIB1 protein stability. Since the stability of AIB1 is known to be regulated by proteasome-mediated degradation, the effect of overexpression of PTEN on the stability of AIB1 was further examined in the absence or presence of MG132, a proteasome inhibitor. The result showed that in the presence of MG132, the levels of AIB1 protein were similar between cells that overexpressed PTEN and cells that did not overexpress PTEN (wild-type or mutant) (Figure 1C), suggesting that MG132 could inhibit the proteasome-dependent degradation of AIB1 promoted by PTEN.

We next examined whether PTEN could affect the level of endogenous AIB1 protein. Expression of endogenous PTEN in MCF-7 cells was knocked down by small interfering RNA (siRNA) and the level of endogenous AIB1 protein was examined. As shown in Figure 1D, knockdown of PTEN increased the level of AIB1 protein without any change in its mRNA level (Figure 1E). The half-life of AIB1 in MCF-7 cells overexpressing wild-type or mutant PTEN was determined after the cells were treated with cycloheximide, an inhibitor of protein biosynthesis. Both wild-type and mutant PTEN reduced the stability of AIB1 (Figure 1F) through increasing its ubiquitination (Figure 1G), but wild-type PTEN appeared to exert a stronger effect.

**Figure 2:**
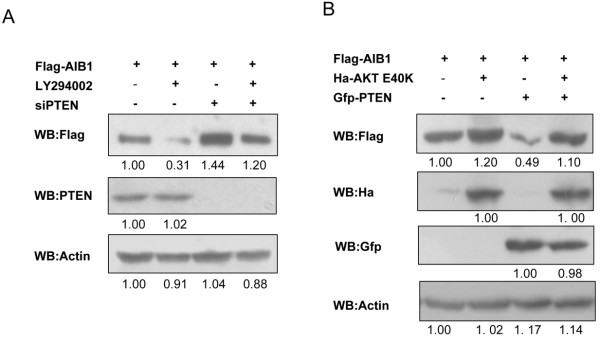
**Reduction of AIB1 protein level by PTEN via PI3K/AKT signaling pathway. (A)** COS-7 cells transfected with Flag-tagged AIB1 and siRNA against PTEN were treated without or with 10 μM LY294002 for 12 h, and then subjected to western blot analysis with the indicated antibodies. **(B)** COS-7 cells transfected with Flag-tagged AIB1 alone or together with Gfp-tagged PTEN or/and Ha-tagged AKT (E40K), and then subjected to western blot analysis with the indicated antibodies.

It is generally believed that PTEN exerts its tumor suppression effect through regulating the PI3K/AKT pathway. This could mean that PTEN might reduce the level of AIB1 protein through inhibiting the PI3K/AKT pathway. Indeed, treatment of COS-7 cells with LY294002 (a specific PI3K inhibitor) resulted in reduced level of AIB1 protein, while knockdown of PTEN had the opposite effect (Figure 2A). On the other hand, overexpression of a constitutively active form of AKT (E40K) resulted in increased level of AIB1 protein in the cells, even when PTEN was also overexpressed (Figure 2B). The data were consistent with our expectation that PTEN could regulate the protein level of AIB1 through interfering with the PI3K/AKT signaling pathway, and this was consistent with the work of Ferrero et al. who showed that the PI3K/AKT pathway can promote the stability of AIB1 [35]. Taken together, these results suggested that PTEN could promote the proteasome-mediated degradation of AIB1, and this process also occurred regardless of whether the phosphatase activity of PTEN was functional or not.

### PTEN can interact with AIB1 through its phosphatase domain

**Figure 3:**
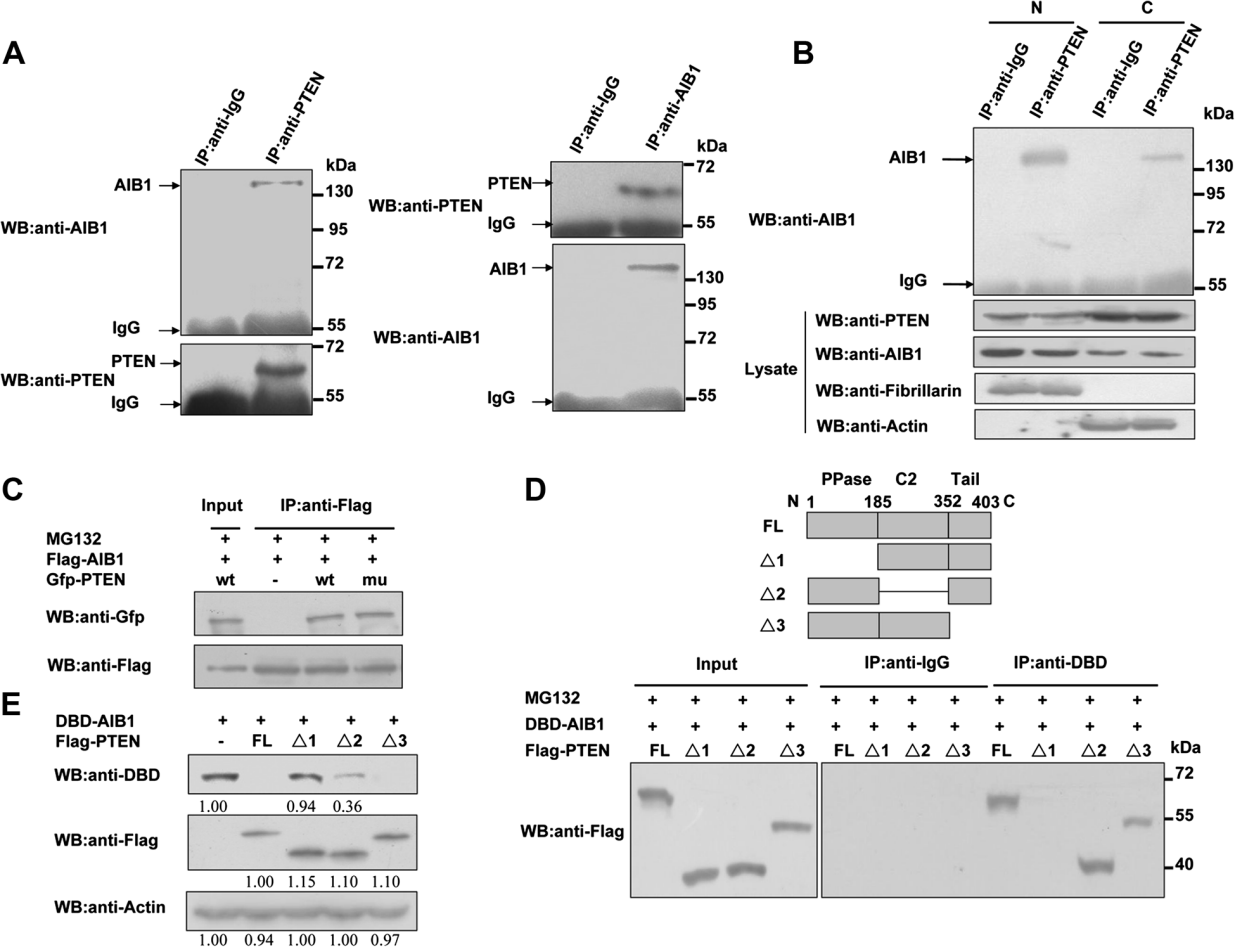
**Interaction between PTEN and AIB1. (A)** MCF-7 cells were treated with 10 μM MG132 for 8 h, and then subjected to immunoprecipitation with anti-PTEN antibody followed by western blot analysis with anti-AIB1 antibody or vice versa. Immunoprecipitation carried out with anti-IgG antibody was used as control. **(B)** MCF-7 cells were treated with 10 μM MG132 for 8 h and then harvested for the preparation of nuclear and cytosolic extracts. These extracts were subjected to immunoprecipitation with anti-IgG or -PTEN antibody followed by western blot analysis with anti-AIB1 antibody. **(C)** COS-7 cells transfected with Flag-tagged AIB1 and Gfp-tagged wt or mu PTEN were treated with 10 μM MG132 for 8 h. The cells were then harvested and subjected to immunoprecipitation with anti-Flag antibody followed by western blot analysis with anti-Gfp antibody. **(D)** COS-7 cells were transfected with Gal4-DBD-tagged AIB1 and Flag-tagged PTEN full-length (FL), PTEN Δ1, PTEN Δ2 or PTEN Δ3 and then treated with 10 μM MG132 for 8 h. The cells were harvested and subjected to immunoprecipitation with anti-IgG or -DBD antibody followed by western blotting with anti-Flag antibody. A Clean-Blot IP Detection Reagent (HRP) from Thermo Scientific which eliminates the detection-interference of IP antibodies was used as secondary antibody. **(E)** COS-7 cells were transfected with Gal4-DBD-tagged AIB1 alone or together with Flag-tagged PTEN FL, PTEN Δ1, PTEN Δ2 or PTEN Δ3 and then subjected to western blot analysis with the indicated antibodies.

As the mutant PTEN G129R could also reduce the level of AIB1 protein (Figure 1A), PTEN and AIB1might interact with each other, and such an interaction might play a role in affecting the level of AIB1 protein. To investigate this possibility, MCF-7 cells were treated with MG132 to prevent the degradation of AIB1, and then immunoprecipitated with anti-PTEN antibody followed by western blot with anti-AIB1 antibody. AIB1 was found to co-precipitate with PTEN, indicating a positive interaction between these two proteins (Figure 3A, left panel). The same result was also obtained when the cell extract was immunoprecipitated with anti-AIB1 antibody followed by western blot with anti-PTEN antibody, thus further confirming the interaction between these two proteins (Figure 3A, right panel). Moreover, nuclear and cytosolic extracts of MCF-7 cells were subjected to immunoprecipitation with anti-IgG or -PTEN antibody followed by western blot with anti-AIB1 antibody. The result showed that the interaction between PTEN and AIB1 occurred both in the cytoplasm and nucleus, but the majority seemed to be in the nucleus (Figure 3B). Similar immunoprecipitation experiments were then carried out using COS-7 cells that were transfected with Flag-tagged AIB1 and Gfp-tagged wild-type or mutant PTEN, followed by treatment with MG132. Extracts prepared from these cells were immunoprecipitated with anti-Flag antibody followed by western blot with anti-Gfp antibody. Both wild-type and mutant PTEN were immunoprecipitated by anti-Flag antibody, demonstrating that the lack of phosphatase activity in PTEN did not affect its interaction (or binding) with AIB1 (Figure 3C).

In order to map the region of PTEN that might interact with AIB1, COS-7 cells were transfected with Gal4-DBD-tagged AIB1 together with Flag-tagged full-length PTEN (PTEN FL) or mutant PTEN having deletion in the PPase (PTEN Δ1), C2 (PTEN Δ2) or Tail domain (PTEN Δ3). The transfected cells were then treated with MG132 before subjecting to immunoprecipitation carried out with anti-DBD antibody, followed by western blot with anti-Flag antibody. No band was detected for the extract prepared from cells transfected with the mutant PTEN Δ1 (Figure 3D), suggesting that the PPase domain was necessary for PTEN to interact with AIB1. We also examined what effect these different truncated forms of PTEN might have on the level of AIB1 protein in the cell. As shown in Figure 3E, PTEN Δ1 could not reduce the level of AIB1, indicating that the PPase domain of PTEN was necessary for PTEN to interact with AIB1 that could lead to the loss of AIB1 protein. PTEN Δ2 caused substantial reduction in the level of AIB1 protein whereas PTEN Δ3 yielded the same result as PTEN FL. This showed that in addition to the PPase domain, which was the major domain responsible for the loss of AIB1 caused by PTEN, the C2 domain also played a role in PTEN-mediated regulation of AIB1.

### PTEN interacts with Fbw7α and increase the ubiquitination of AIB1

**Figure 4:**
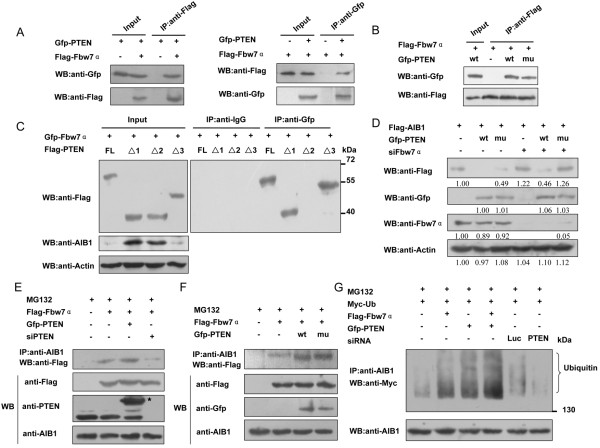
**Effect of PTEN on the interaction between Fbw7**α **and AIB1. (A)** COS-7 cells transfected with Flag-tagged Fbw7α and Gfp-tagged PTEN were subjected to immunoprecipitation with anti-Flag antibody followed by western blot with anti-Gfp antibody or vice versa. **(B)** COS-7 cells transfected with Flag-tagged Fbw7α and Gfp-tagged wt or mu PTEN were subjected to immunoprecipitation with anti-Flag antibody followed by western blot with anti-Gfp antibody. **(C)** COS-7 cells transfected with Gfp-tagged Fbw7α and Flag-tagged PTEN FL, PTEN Δ1, PTEN Δ2 or PTEN Δ3 were collected and then subjected to immunoprecipitation with anti-Gfp antibody, followed by western blot analysis with anti-Flag antibody. A Clean-Blot IP Detection Reagent (HRP) from Thermo Scientific which eliminates the detection-interference of IP antibodies was used as secondary antibody. **(D)** COS-7 cells were transfected with Flag-tagged AIB1 and Gfp-tagged wt or mu PTEN and with or without siFbw7α. Cells were collected and subjected to western blot analysis with the indicated antibodies. **(E)** 293T cells were transfected with Flag-tagged Fbw7α with or without Gfp-tagged PTEN or siPTEN as indicated, and then treated with 10 μM MG132 for 8 h. Cells were collected and then subjected to immunoprecipitation with anti-AIB1 antibody followed by western blot with anti-Flag antibody. ^★^ indicates overexpressed Gfp-tagged PTEN. **(F)** 293T cells were transfected with Flag-tagged Fbw7α with or without wt or mu PTEN as indicated, and then treated with 10 μM MG132 for 8 h. The cells were harvested and subjected to immunoprecipitation with anti-AIB1 antibody followed by western blot with anti-Flag antibody. **(G)** 293T cells transfected with various combinations of different constructs as indicated were treated with 10 μM MG132 for 8 h. The cells were collected and subjected to immunoprecipitation with anti-AIB1 antibody followed by western blot analysis with anti-Myc antibody.

As PTEN can increase the interaction between APC and CDH1, and therefore enhance the activity of the APC-CDH1 complex (an E3 ubiquitin ligase) and promote the degradation of its target proteins, we speculated that PTEN might also down-regulate the level of AIB1 protein through affecting its E3 ubiquitin ligase, Fbw7α. Indeed, by subjecting the extract from COS-7 cells that had been transfected with Gfp-tagged PTEN and Flag-tagged Fbw7α to immunoprecipitation with anti-Flag antibody followed by western blot with anti-Gfp antibody, a clear band corresponded to Flag-Fbw7α was detected (Figure 4, left panel), suggesting a positive interaction between PTEN and Fbw7α. The same result was obtained from a reciprocal immunoprecipitation experiment (Figure 4A, right panel). Both wild-type and mutant PTEN were precipitated by Fbw7α, indicating that the interaction between PTEN and Fbw7α did not require the phosphatase activity of PTEN (Figure 4B). By performing the same experiment for the different truncated PTEN mutants, the region of PTEN that interacted with Fbw7α was mapped to the C2 domain (Figure 4C, top panel). Since overexpression of PTEN Δ1 (deficient in AIB1-interacting domain) and Δ2 (deficient in Fbw7α-interacting domain) along with Fbw7α could not reduce the protein level of endogenous AIB1 as could PTEN FL and PTEN Δ3 (Figure 4C, bottom panel), it suggested that both PTEN-AIB1 and PTEN-Fbw7α interactions were important for the PTEN-mediated degradation of AIB1. As shown in Figure 4D, loss of AIB1 in COS-7 cells caused by overexpression of wild-type PTEN or phosphatase activity-deficient mutant PTEN was reduced when Fbw7α was knocked down. This showed that Fbw7α could facilitate PTEN-mediated degradation of AIB1, irrespective of whether the degradation stemmed from the phosphatase activity of PTEN or not, and suggested that the role of Fbw7α might be to facilitate the degradation of AIB1. So we speculated that PTEN might affect the interaction between AIB1 and Fbw7α. As shown in Figure 4E, overexpression of PTEN and Fbw7α resulted in the highest level of Fbw7α being pulled down by AIB1 compared to overexpression of Fbw7α alone, while knockdown of PTEN coupled with overexpression of Fbw7α resulted in very low level of pulled-down Fbw7α, suggesting that PTEN probably increased the interaction between Fbw7α and AIB1. Moreover, overexpression of the mutant PTEN deficient in phosphatase activity also resulted in similar increase in pulled-down Fbw7α as obtained with overexpression of wild-type PTEN (Figure 4F). Next, we examined the effect of PTEN and Fbw7α on the ubiquitination of AIB1. As shown in Figure 4G, PTEN and Fbw7α both increased the ubiquitination of AIB1, with the extent of ubiquitination being stronger when both PTEN and Fbw7α were overexpressed. In contrast, knockdown of PTEN reduced the ubiquitination of AIB1. Taken together, these results suggested that PTEN could increase the interaction between AIB1 and Fbw7α, therefore, promoting the ubiquitin-mediated degradation of AIB1 through Fbw7α.

### PTEN suppresses the transcriptional activity of AIB1

**Figure 5:**
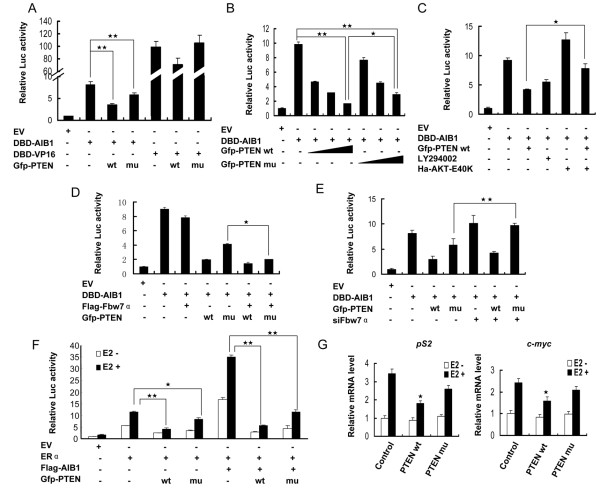
**Effect of PTEN on the transcriptional activity of AIB1. (A)** COS-7 cells were transfected with Gal4-luc reporter construct and Gal4-DBD-tagged AIB1 or VP16 together with wt or mu PTEN. pcDNA3.1(−)/pcGal4-DBD vector was used as empty vector (EV). Twenty four hours after transfection, the cells were collected and subjected to luciferase activity assay. **(B)** COS-7 cells were transfected with Gal4-luc reporter construct and Gal4-DBD-tagged AIB1 together with increasing dosages (10, 100, 300 ng) of Gfp-tagged wt or mu PTEN. pcDNA3.1(−)/pcGal4-DBD vector was used as EV. Twenty four hours after transfection, the cells were collected and subjected to luciferase activity assay. **(C)** COS-7 cells were transfected with Gal4-luc reporter construct and Gal4-DBD-tagged AIB1 together with or without Gfp-tagged PTEN. pcDNA3.1(−)/pcGal4-DBD vector was used as EV. The cells were treated with or without 10 μM LY294002 for 12 h, and then subjected to luciferase activity assay. **(D & E)** COS-7 cells were transfected with Gal4-luc reporter construct and Gal4-DBD-tagged AIB1 together with various combinations of other constructs as indicated. pcDNA3.1(−)/pcGal4-DBD vector was used as EV. Twenty four hours after transfection, the cells were harvested and subjected to luciferase activity assay. **(F)** MCF-7 cells were transfected with ERE-luc reporter and ERα constructs along with other plasmids as indicated. pcDNA3.1(−) vector was used as EV. Eight hours after transfection, the cells were switched to phenol red-free medium containing 10% charcoal-dextran-treated fetal bovine serum for 16 h followed by treatment with or without 10 nM E2 for another 16 h, and then subjected to luciferase activity assay. **(G)** MCF-7 cells were transfected with Gfp-tagged wt or mu PTEN. Eight hours after transfection, the cells were switched to phenol red-free medium containing 10% charcoal-dextran-treated fetal bovine serum for 16 h followed by treatment with or without 10 nM E2 for another 16 h. The cells were then subjected to real-time PCR to measure the mRNA level of *pS2* and *c-myc*. Each bar represents the mean ± S.D. from three independent experiments. *, *P* < 0.05; **, *P* < 0.01.

As PTEN was able to reduce the level of AIB1 protein, we examined whether this will lead to change in the transcriptional activity of AIB1. The effect of PTEN on the transcriptional activity of AIB1 was first determined using Gal4 promoter-driven luciferase (Gal4-luc) construct as a reporter gene. COS-7 cells were transfected with Gal4-luc construct, Gal4-DBD-tagged AIB1 and either Gfp-tagged wild-type or mutant PTEN, and the reporter activity of these cells was measured. Gal4-DBD-tagged VP16, a potent transcriptional activator was used as a control. Both wild-type and mutant PTEN suppressed the transcriptional activity of AIB1 although the mutant exhibited a weaker effect (Figure 5A). The suppression of AIB1 transcriptional activity by PTEN was also dose-dependent (Figure 5B). Wild-type PTEN also suppressed the transcriptional activity of VP16, but to a much lesser extent compared to the suppression of the transcriptional activity of AIB1 (~25% versus ~57%). In contrast, mutant PTEN deficient in phosphatase activity was not able to suppress the transcriptional activity of VP16, suggesting that suppression of AIB1 transcriptional activity by PTEN was rather specific (Figure 5A). These results corresponded to the reduction in the level of AIB1 protein detected by western blot (Figure 1A), and therefore reaffirmed that PTEN may suppress the transcriptional activity of AIB1 regardless of whether the phosphatase activity of PTEN was present or absent. Moreover, LY294002 also reduced AIB1 transactivation, whereas AKT E40K increased AIB1 transactivation, and consequently compensated for the PTEN-induced inhibition of AIB1 transactivation (Figure 5C). This suggested that PTEN could regulate the function of AIB1 through inhibiting the PI3K/AKT signaling pathway, and to achieve this, PTEN requires a functional phosphatase activity. Overexpression of Fbw7α also enhanced the inhibition of AIB1 transactivation mediated by wild-type and mutant PTEN, especially the inhibition mediated by the mutant (Figure 5D). Knockdown of Fbw7α by siRNA blocked the inhibitory effect exerted by PTEN on AIB1 transactivation, especially the effect exerted by the mutant (Figure 5E). Taken together these results suggested that Fbw7α played an important role in the PTEN-mediated suppression of AIB1 transactivation that did not require the functional phosphatase activity of PTEN.

As AIB1 is a major coactivaor of ERα, we also examined whether PTEN could affect the transcriptional activity of ERα through its influence on AIB1 as determined by the estrogen responsive element luciferase (ERE-luc) reporter gene. As shown in Figure 5F, in the presence of E2 treatment, ERE-luc activity was highest when the cells overexpressed ERα and AIB1. However, if these cells also overexpressed PTEN, the level of ERE-luc activity was significantly reduced, with about 84% reduction occurred when the cells overexpressed wild-type PTEN or about 68% reduction when the cells overexpressed the mutant PTEN G129R. Although the level of ERE-luc activity in the absence of AIB1 overexpression was only about 31% the level achieved in the presence of AIB1 overexpression, marked reduction (~65%) still occurred when the cells overexpressed wild-type PTEN without overexpressing AIB1. This suggested that loss of ERE-luc activity could be due to direct suppression of ERα or AIB1 or both ERα and AIB1 by PTEN. In addition, this suppression was not affected by the absence of E2 since PTEN is not a hormone-activating protein. Indeed, PTEN could both inhibit the transcriptional activity of AIB1 in the absence and presence of E2 treatment (data not shown). Furthermore, we examined what effect overexpression of wild-type or mutant PTEN might have on the expression of two ERα-AIB1 target genes (*pS2* and *c-myc*) in MCF-7 cells. The mRNA levels of *pS2* and *c-myc* were reduced by about 47% and 34%, respectively, when the cells overexpressed wild-type PTEN. However, when the cells overexpressed the mutant PTEN, the levels of *pS2* and *c-myc* were reduced by about 24% and 14%, respectively (Figure 5G). These results corresponded to those obtained from reporter gene assays, suggesting that PTEN could regulate activities of ERα and AIB1 in a manner that is not entirely dependent on its phosphatase activity.

### PTEN inhibits the oncogenic function of AIB1

**Figure 6:**
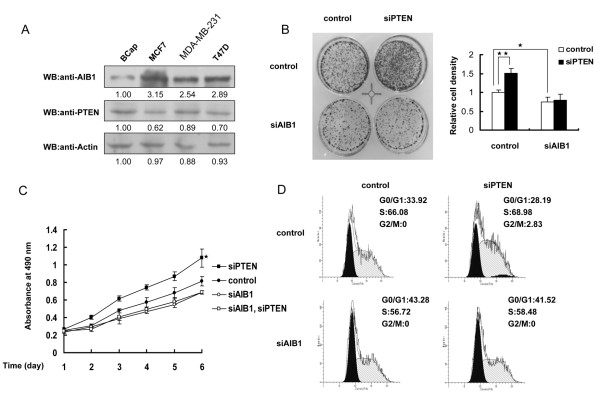
**Effect of PTEN on AIB1-induced cell proliferation. (A)** Western blot analysis comparing the endogenous AIB1 and PTEN protein levels in several breast cancer cells. **(B)** MCF-7 Cells transfected with siRNA against AIB1 or/and PTEN were stained with crystal violet after 8 days of growth (left panel). The right graph shows the relative cell density obtained from three plates estimated by the software Imagepro-pus. Each bar represents the mean ± S.D. from three plates *, *P* < 0.05; **, *P* < 0.01. **(C)** MCF-7 cells were transfected with siRNA against AIB1 or/and PTEN for different time points and the cell proliferation status was assessed by MTT assay. Each bar represents the mean ± S.D. from five independent experiments. *, *P* < 0.05 (control versus cells with knockdown of PTEN). **(D)** Flow cytometry analysis on cell-cycle distribution of MCF-7 cells after 2 days of transfection.

We examined the protein levels of endogenous AIB1 and PTEN in various breast cancer cell lines. As shown in Figure 6A, the endogenous levels of AIB1 and PTEN proteins appeared to have a reverse relationship, with higher level of AIB1 protein correlated with lower level of PTEN protein. The biological consequences of PTEN and AIB1 in MCF-7 cells were also assessed. As shown in Figure 6B, MCF-7 cells in which PTEN was knocked down exhibited greater number of cells than MCF-7 cells without knockdown of PTEN. On the other hand, knockdown of AIB1 produced no obvious difference in the number of cells between MCF-7 cells with or without knockdown of PTEN. Similarly, cells with PTEN knockdown grew faster than control cells. However when AIB1 was also knocked down, knockdown of PTEN had no effect on cell proliferation (Figure 6C). The effect of PTEN on the cell-cycle was also investigated using cells with or without AIB1 knockdown. Knockdown of PTEN had no effect on the percentage of cells in the G0/G1 phase when AIB1 was also knocked down (Figure 6D). Taken together, these results suggested that PTEN could regulate the growth of MCF-7 cells through its modulation of AIB1.

## Discussion

PTEN is well characterized as a tumor suppressor that negatively regulates the PI3K/AKT pathway-driven tumor progression, and the phosphatase activity of PTEN is vital for this function [36-39]. However there are more and more evidences suggesting that PTEN can also exert its function without its phosphatase activity [40,41]. For example, PTEN can physically interact with p53 and regulate the protein stability and transcriptional activity of p53 [42,43]. PTEN also forms a complex with p300 to maintain a high level of acetylation of p53 in response to DNA damage [44]. PTEN directly interacts with androgen receptor (AR) to inhibit its nuclear translocation and promote its degradation, resulting in the suppression of AR transactivation and AR-mediated apoptosis [45].

Here we showed that PTEN increased the ubiquitin-dependent degradation of AIB1, therefore reducing the protein but not mRNA level of AIB1. Both wild-type PTEN and to a lesser extent, the G129R PTEN mutant deficient in both lipid and protein phosphatase activities were able to reduce the level of AIB1 protein when overexpressed in COS-7 cells (Figure 1A). LY294002 also reduced the level of AIB1 protein, whereas E40K, the constitutively active form of AKT, appeared to counteract the effect of PTEN and preserved the level of AIB1 protein when both E40K and PTEN were overexpressed in the cell (Figure 2A and B), which suggested that PTEN could regulate the level of AIB1 protein through inhibiting step(s) within the PI3K/AKT signaling pathway. These results were consistent with the result obtained with reporter gene assay (Figure 5A-C). This could mean that PTEN might regulate the function of AIB1 through decreasing its protein stability. Since both wild-type and the phosphatase activity-deficient PTEN mutant were able to affect the level of AIB1 protein and hence its transcriptional activity, it suggested that PTEN might also regulate AIB1 via another mechanism in addition to that which depends on its phosphatase activity. This mechanism may stem from PTEN playing a structural role, such as stabilizing the protein complex, thereby making AIB1 more readily for ubiquitination. We speculated that there could be two ways in which PTEN might regulate the protein stability and transcriptional activity of AIB1: 1) by inhibiting the PI3K/AKT signaling pathway whereby the phosphatase activity of PTEN is essential; and 2) by an alternative mechanism that does not require the phosphatase activity of PTEN. The lack of effect exerted by the phosphatase activity-deficient mutant PTEN on the protein stability (supported by weaker level of ubiquitination) and transcriptional activity of AIB1 compared to wild-type PTEN was consistent with its inability to inhibit the PI3K/AKT signaling pathway.

In ubiquitin-dependent protein degradation, the ubiquitin must be attached to the target protein by an E3 ubiquitin ligase before it is targeted for degradation via the 26S proteosome. Previous study has shown that integration site 6 (Int6), which is required for the assembly of a functional proteasome machinery, can regulate the level of AIB1 protein, probably through mediating the interaction between AIB1 and Fbw7α [46]. We speculated that PTEN may also reduce the level of AIB1 protein through regulating the interaction between AIB1 and Fbw7α. Our data demonstrated that interaction between PTEN and AIB1 occurred at the PPase domain of PTEN, whereas interaction between PTEN and Fbw7α occurred at the C2 domain of PTEN. As well as wild-type PTEN, the mutant PTEN G129R also facilitated the interaction between AIB1 and Fbw7α (Figure 4F). This again suggested that PTEN might play a structural role, such as by acting as a bridge connecting AIB1 and Fbw7α, helping to bring the two proteins into close proximity favorable for Fbw7α to act on AIB1. Although we have no direct evidence to show that such AIB1/PTEN/Fbw7α complex exists endogenously, the data we obtained from immuoprecipitation experiments were consistent with the likelihood of the existence of such complex. Furthermore, such a role of PTEN is also consistent with its regulation of AIB1 that is not based on its phosphatase activity. This was confirmed by the reporter gene assays which showed that overexpression of Fbw7α along with the mutant PTEN G129R in COS-7 cells strongly inhibited the transcriptional activity of AIB1, but when Fbw7α was knocked down, overexpression of the mutant PTEN alone failed to inhibit the transcriptional activity of AIB1 (Figure 5D and E), meaning that Fbw7α is necessary for PTEN to fulfill its structural role in the regulation of AIB1.

AIB1 has an important role in promoting cell growth and in inhibiting apoptosis. Overexpression of AIB1 in prostate cancer cell lines results in increased cell size and induction of cell growth [47], whereas knockdown of AIB1 expression by siRNA blocks estradiol-stimulated cell proliferation [48]. Since PTEN could act as a negative regulator of AIB1, PTEN would be expected to play a role in AIB1-mediated cell proliferation. Indeed, knockdown of PTEN, which reduced the loss of AIB1, and therefore alleviated the suppression on AIB1-promoted cell proliferation as seen with increased cell growth relative to control (no knockdown of PTEN and AIB1), whereas knockdown of both PTEN and AIB1 reduced cell growth relative to control, and was to the same extent as when only AIB1 was knocked down (Figure 5B and C). This suggested that PTEN might indeed act as a tumor suppressor to suppress the oncogenic function of AIB1.

## Conclusions

We showed here for the first time that the function of AIB1 is subject to negative regulation by PTEN. PTEN could regulate the ubiquitination and hence the protein stability of AIB1. Although this process involved the phosphatase activity of PTEN and PI3K/AKT signaling pathway, we have demonstrated an alternative mechanism by which PTEN might regulate the stability and hence activity of AIB1, one in which the interaction between PTEN and Fbw7α appeared to be an important contributing factor. The effect of PTEN-mediated regulation of AIB1 activity was confirmed by an AIB1 reporter gene assay and AIB1-mediated ERα reporter gene assay, which showed that PTEN might also regulate the activity of other transcription factors through AIB1. The molecular mechanism by which the regulation of AIB1 by PTEN could lead to change in the activity of downstream genes and its implication in tumorigenesis will be a subject of further study.

## Materials and methods

### Cell culture and plasmids

MCF-7 and COS-7 cells had been used in our previous study [49]. 293T, BCap and MDA-MB-231 cells obtained from ATCC were maintained in Dulbecco’s modified Eagle’s medium (DMEM) supplemented with 10% fetal bovine serum (Hyclone) and penicillin-streptomycin (100 U/ml penicillin and 0.1 mg/ml streptomycin). T47D cells obtained from ATCC were maintained in Roswell Park Memorial Institute (RPMI) 1640 medium supplemented with 10% fetal bovine serum, penicillin-streptomycin and insulin (5g/ml). Cells were incubated at 37°C in a humidified incubator with 5% CO_2_.

pcDNA3/Gfp-PTEN was a gift kindly provided by Dr. Alonzo H. Ross (University of Massachusetts). Gfp-tagged PTEN G129R was constructed by mutating Gly129 to Arg using the Quick-Change site-directed mutagenesis kit (Stratagene, La Jolla, CA). pcDNA3/Gfp-PTEN was used as a template and the mutagenesis was performed according to the instruction of the manufacturer. Flag-tagged full-length and truncated PTEN (Δ1, Δ2, and Δ3) were provided by Dr. Shiaw-Yih Lin (University of Texas). p3xFlag-CMV10/AIB1 was provided by Dr. Anna T. Riegel (Georgetown University Medical Center). pcDNA3.1(−)/pcGal4-DBD-AIB1 was prepared by cloning the full-length AIB1 gene into pcDNA3.1(−)-Gal4-DBD. pcDNA3.1/ERE luciferase reporter was provided by Dr. Carolyn L. Smith (Baylor College of Medicine). pCMV/Ha-AKT(E40K) was provided by Dr. Jaime Font de Mora (University Hospital of Salamanca). p3xFlag-CMV7.1/Fbw7α was provided by Dr. Deanna M. Koepp (University of Minnesota-Twin Cities). pEgfp-C1/Fbw7α was prepared by cloning the full-length Fbw7α into pEgfp-C1. pSURE/siPTEN was obtained from Dr. Baiqu Huang (Northeast Normal University). pRNAT-U6.1/siAIB1 and pRNAT-U6.1/siFbw7 was constructed with these target sequences: 5^′^-TCCTGCAGTGTATAGTATG-3^′^ for AIB1 and 5^′^-GGGCAACAACGACGCCGAA-3^′^ for Fbw7.

### Antibodies and reagents

Rabbit polyclonal anti-Flag, anti-Gfp, anti-AIB1, anti-PTEN, anti-DBD and mouse monoclonal anti-Actin antibody were obtained from Santa Cruz Biotechnology (Santa Cruz, CA). Rabbit polyclonal anti-Myc and mouse monoclonal anti-Flag (M2) antibodies were purchased from Sigma. Cycloheximide was obtained from Sigma; MG132 was obtained from Merck and LY294002 was obtained from Selleck.

### Reporter assays

To determine the transcriptional activity of AIB1, COS-7 cells were grown in 24-well plates and transfected with the appropriate plasmids using Lipofectamine 2000 (Invitrogen). The transfection procedure was carried out according to the instruction of the manufacturer. Twenty four hours after transfection, the cells were rinsed with PBS and subjected to luciferase and Renilla activity assays using a dual luciferase kit (Promega, Madison, WI).

To determine the transcriptional activity of ERα, MCF-7 cells were plated in 24-well plates and transfected with the appropriate plasmids. Eight hours after transfection, the cells were switched to phenol red-free medium containing 10% charcoal-dextran-treated fetal bovine serum for 16 h followed by treatment with or without 10 nM 17-estradiol (E2) for another 16 h. The cells were then harvested, and subjected to luciferase and Renilla activity assays.

### Western blot, immunoprecipitation

MCF-7 and COS-7 cells were lysed in a cold buffer containing 50 mM Tris–HCl (pH 8.0), 150 mM NaCl, 0.1% SDS, 1% NP-40, 0.5% sodium deoxycholate and protease inhibitor mixture (Roche Applied Science), and then subjected to SDS-PAGE. Proteins in the gel were transferred to PVDF membrane (Millipore) and probed with the specified primary antibody, followed by the appropriate secondary antibody, and then visualized using the enhanced chemiluminescence detection reagents (Thermo) according to the manufacturer’s instructions.

Immunoprecipitation experiments were carried out using COS-7, 293T and MCF-7 cell extracts. The cells were lysed in cold buffer containing 50 mM Tris–HCl (pH 7.4), 150 mM NaCl, 1% NP-40 and protease inhibitor mixture. The cell lysate was centrifuged at 12000 × *g/*4°C for 10 min, and the supernatant was incubated with protein A-Sepharose (Amersham Biosciences) or protein G-Sepharose (Santa Cruz, CA) at 4°C for 1 h. It was then centrifuged at 5000 × *g/*4°C for 10 min and the supernatant was incubated with fresh protein A- or protein G-Sepharose and the desired antibody at 4°C for overnight. After that, the pellet was collected by centrifugation at 5000 × *g/*4°C and washed twice with Wash Buffer I (50 mM Tris–HCl [pH 7.5], 150 mM sodium chloride, 1% NP-40 and 0.05% sodium deoxycholate) and once with Wash Buffer II (50 mM Tris–HCl [pH 7.5], 500mM sodium chloride, 0.1% NP-40, and 0.05% sodium deoxycholate). After washing, it was resuspended in 1 × SDS-PAGE loading buffer, heated at 100°C for 5 min and then resolved in 8% or 10% gel. The proteins in the gel were transferred to PVDF membrane and subjected to western blot as described above.

### Real-time RT-PCR

MCF-7 cells were plated in 6-well plates and transfected with the appropriate plasmids. Eight hours after transfection, the cells were switched to phenol red-free medium containing 10% charcoal-dextran-treated fetal bovine serum for 16 h followed by treatment with or without 10 nM E2 for another 16 h. Total RNA was isolated from the cells using TRIzol reagent (Takara) according to the manufacturer’s instruction, and then subjected to reverse transcription with oligo(dT)15. *pS2, c-myc* and *GADPH* (as an internal control) mRNA were quantitated by real-time PCR using Corbett Research RG 3000 analyzer, RealMasterMix (SYBR Green) (TIANGEN BIOTECH, BEIJING). The following primers sequences were used: *pS2*, 5^′^-TTCTATCCTAATACCATCGACG-3^′^ and 5^′^-TTTGAGTAGTCAAAGTCAGAGC-3^′^; *c-myc*: 5^′^-TCCACACATCAGCACAACTACG-3^′^ and 5^′^-CACTGTCCAACTTGACCCTCTTG-3^′^; *GADPH*,5^′^-GGGTGTGAACCATGAGAAGT-3^′^ and 5^′^-GACTGTGGTCATGAGTCCT-3^′^. The mRNA levels of *pS2 and c-myc* were normalized to *GAPDH*, which served as the endogenous control. Each gene was measured in triplicate.

### Cell proliferation assays

MTT and Flow Cytometry assays were performed as previously desecribed [50]. For crystal violet staining, 5000 MCF-7 cells were transfected with the appropriate plasmids and then transferred into 35 mm plate in the presence of DMEM. After eight days of growth, the cells were stained with crystal violet (Sigma) for 30 min at room temperature.

### Statistical analysis

All data were analysed by ANOVA [51] when necessary. Data are given as means ± SDs, and significance was considered at either *P* value < 0.05 or 0.01 level.

## Abbreviations

PTEN: Phosphatase and tensin homologue deleted on chromosome 10; AIB1: Amplified in breast cancer 1; Fbw7α: F-box and WD repeat domain-containing 7 alpha; PIP3: Phosphoinositide-3,4,5-triphos-phate; CENP-C: Centromere protein C; APC/C: Anaphase-promoting complex/cyclosome; CDH1: Cadherin 1; ERα: Estrogen receptor alpha; EGFR: Epidermal growth factor receptor; IGF: Insulin-like growth factor; PDXP: Pyridoxal phosphate phosphatase; PP1: Protein phosphatase 1; PP2A: Protein phosphatase 2A; E6-AP: E6-associated protein; SPOP: Speckle-type POZ protein; siRNA: Small interfering RNA; Int6: Integration site 6.

## Competing interests

The authors declare that they have no competing interests.

## Authors’ contributions

HW conceived and designed the experiments. CY performed the experiments. CY analyzed the data. CY, SL, MW, AKC, YL, FZ, LX, LH, DW and SL contributed reagents/materials/analysis tools. HW wrote the paper. All authors read and approved the final manuscript.
